# Supplementary material for: GrapHiC: An Integrative Graph Based Approach for Imputing Missing Hi-C Reads
Source: IEEE Trans Comput Biol Bioinform. Author manuscript; Available in PMC 2025 Aug 15. (PMC12034241; doi:10.1109/TCBB.2024.3477909)
Supplement: supp1-3477909 [file NIHMS2072650-supplement-supp1-3477909.pdf]

Component		Layer	Input Shape	Output Shape
**Graph Encoder**		TransformerConv(13, 32, heads=4)	(-1, 256, 13)	(-1, 256, 32)
		Linear(in_features=128, out_features=32, bias=True)	(-1, 256, 32)	(-1, 256, 32)
		GraphNorm(32)	(-1, 256, 32)	(-1, 256, 32)
**Graph Decoder**	**InnerProductDecoder**	InnerProduct	(-1, 256, 32)	(-1, 1, 256, 256)
		Conv2d(1, 32, kernel_size=(3, 3), stride=(1, 1), padding=(1, 1))	(-1, 256, 256)	(-1, 32, 256, 256)
	**DownBlock**	GroupNorm(8, 128, eps=1e-05, affine=True)	(-1, 32, 256, 256)	(-1, 32, 256, 256)
		Conv2d(128, 64, kernel_size=(3, 3), stride=(1, 1), padding=(1, 1))	(-1, 32, 256, 256)	(-1, 32, 256, 256)
		GroupNorm(8, 64, eps=1e-05, affine=True)	(-1, 32, 256, 256)	(-1, 32, 256, 256)
		Conv2d(64, 64, kernel_size=(3, 3), stride=(1, 1), padding=(1, 1))	(-1, 32, 256, 256)	(-1, 32, 256, 256)
		Conv2d(32, 32, kernel_size=(3, 3), stride=(2, 2), padding=(1, 1))	(-1, 32, 256, 256)	(-1, 32, 128, 128)
	**DownBlock**	GroupNorm(8, 128, eps=1e-05, affine=True)	(-1, 32, 128, 128)	(-1, 32, 128, 128)
		Conv2d(128, 64, kernel_size=(3, 3), stride=(1, 1), padding=(1, 1))	(-1, 32, 128, 128)	(-1, 32, 128, 128)
		GroupNorm(8, 64, eps=1e-05, affine=True)	(-1, 32, 128, 128)	(-1, 32, 128, 128)
		Conv2d(64, 64, kernel_size=(3, 3), stride=(1, 1), padding=(1, 1))	(-1, 32, 128, 128)	(-1, 32, 128, 128)
		Conv2d(32, 32, kernel_size=(3, 3), stride=(2, 2), padding=(1, 1))	(-1, 32, 128, 128)	(-1, 32, 64, 64)
	**DownBlock**	GroupNorm(8, 128, eps=1e-05, affine=True)	(-1, 32, 64, 64)	(-1, 32, 64, 64)
		Conv2d(128, 64, kernel_size=(3, 3), stride=(1, 1), padding=(1, 1))	(-1, 32, 64, 64)	(-1, 32, 64, 64)
		GroupNorm(8, 64, eps=1e-05, affine=True)	(-1, 32, 64, 64)	(-1, 32, 64, 64)
		Conv2d(64, 64, kernel_size=(3, 3), stride=(1, 1), padding=(1, 1))	(-1, 32, 64, 64)	(-1, 32, 64, 64)
		Conv2d(32, 32, kernel_size=(3, 3), stride=(2, 2), padding=(1, 1))	(-1, 32, 64, 64)	(-1, 64, 32, 32)
	**Middle Block**	SelfAttention()	(-1, 64, 32, 32)	(-1, 64, 32, 32)
	**UpBlock**	Concatenate(Downblock + Previous Block)	(-1, 64, 32, 32)*2	(-1, 128, 32, 32)
		GroupNorm(8, 64, eps=1e-05, affine=True)	(-1, 128, 32, 32)	(-1, 128, 32, 32)
		Conv2d(128, 64, kernel_size=(3, 3), stride=(1, 1), padding=(1, 1))	(-1, 128, 32, 32)	(-1, 64, 32, 32)
		GroupNorm(8, 64, eps=1e-05, affine=True)	(-1, 64, 32, 32)	(-1, 64, 32, 32)
		Conv2d(64, 32, kernel_size=(3, 3), stride=(1, 1), padding=(1, 1))	(-1, 64, 32, 32)	(-1, 32, 32, 32)
		SelfAttention()	(-1, 32, 32, 32)	(-1, 32, 32, 32)
		ConvTranspose2d(32, 32, kernel_size=(4, 4), stride=(2, 2), padding=(1, 1))	(-1, 64, 32, 32)	(-1, 32, 64, 64)
	**UpBlock**	Concatenate(Downblock + Previous Block)	(-1, 32, 64, 64)*2	(-1, 64, 64, 64)
		GroupNorm(8, 64, eps=1e-05, affine=True)	(-1, 64, 64, 64)	(-1, 64, 64, 64)
		Conv2d(64, 32, kernel_size=(3, 3), stride=(1, 1), padding=(1, 1))	(-1, 64, 64, 64)	(-1, 32, 64, 64)
		GroupNorm(8, 32, eps=1e-05, affine=True)	(-1, 32, 64, 64)	(-1, 32, 64, 64)
		Conv2d(32, 32, kernel_size=(3, 3), stride=(1, 1), padding=(1, 1))	(-1, 32, 64, 64)	(-1, 32, 64, 64)
		SelfAttention()	(-1, 32, 64, 64)	(-1, 32, 64, 64)
		ConvTranspose2d(32, 32, kernel_size=(4, 4), stride=(2, 2), padding=(1, 1))	(-1, 32, 64, 64)	(-1, 32, 128, 128)
	**UpBlock**	Concatenate(Downblock + Previous Block)	(-1, 32, 128, 128)*2	(-1, 64, 128, 128)
		GroupNorm(8, 64, eps=1e-05, affine=True)	(-1, 64, 128, 128)	(-1, 64, 128, 128)
		Conv2d(64, 32, kernel_size=(3, 3), stride=(1, 1), padding=(1, 1))	(-1, 64, 128, 128)	(-1, 32, 128, 128)
		GroupNorm(8, 32, eps=1e-05, affine=True)	(-1, 32, 128, 128)	(-1, 32, 128, 128)
		Conv2d(32, 32, kernel_size=(3, 3), stride=(1, 1), padding=(1, 1))	(-1, 32, 128, 128)	(-1, 32, 128, 128)
		SelfAttention()	(-1, 32, 128, 128)	(-1, 32, 128, 128)
		ConvTranspose2d(32, 32, kernel_size=(4, 4), stride=(2, 2), padding=(1, 1))	(-1, 32, 128, 128)	(-1, 32, 256, 256)
	**Final Projection**	GroupNorm(8, 32, eps=1e-05, affine=True)	(-1, 32, 256, 256)	(-1, 32, 256, 256)
		Conv2d(32, 1, kernel_size=(3, 3), stride=(1, 1), padding=(1, 1))	(-1, 32, 256, 256)	(-1, 1, 256, 256)
		Sigmoid()	(-1, 1, 256, 256)	(-1, 1, 256, 256)

TABLE S1
This table provides the detailed breakdown of all the layers in our GrapHiC model.

	Reads	Sparsity	Source
**GRCh38-GM12878-HRC**	6,524,520,477	1	ENCFF555ISR
**GRCh38-GM12878-LRC**	283,697,048	23	ENCFF216ZNY
**GRCh38-K562-HRC**	2,188,905,398	1	ENCFF080DPJ
**GRCh38-K562-LRC**	608,231,511	4	4DNESI7DEJTM

TABLE S2
We add four Hi-C datasets, two from GM12878 and two from K562 that are aligned to the GRCh38 assembly to evaluate how GrapHiC generalizes to different assemblies.

		MSE	SSIM	PCC	HiCRep	GenomeDISCO	HiCSpector	QuASAR-Rep	TAD Boundaries	Chromatin Loops	DNA Hairpins
**GM12878**	**hg19**	**0.0009**	**0.9224**	**0.9797**	**0.8008**	**0.9100**	**0.4614**	**0.8609**	**0.6648**	**0.5216**	**0.5272**
	**grch38**	0.0016	0.9053	0.9544	0.7933	0.8822	0.4367	0.8699	0.6514	0.5119	0.4946
**K562**	**hg19**	0.0047	0.8278	0.8731	0.7088	**0.8470**	**0.3252**	0.7342	0.5388	**0.4522**	0.3703
	**grch38**	**0.0024**	**0.8747**	**0.9262**	**0.7127**	0.7753	0.3152	**0.7848**	**0.5698**	0.4386	**0.4297**

TABLE S3
We compare the performance of GrapHiC trained on hg19 aligned datasets on Hg19 and GRCh38 aligned datasets for GM12878 and K562 cell lines. We observe a minor decrease in the GM12878 cell line scores; we believe this change arises because the GRCh38 GM12878 HRC dataset has a substantially higher number of reads in comparison to Hg19 GM12878 HRC (1.8 billion vs. 6.4 billion reads). The Hi-C contact maps generated by GrapHiC match the feature distribution of the 1.8 billion reads contact map and have a smaller set of features compared to the 6.4 billion reads contact map. This difference manifests as a degradation in scores. Conversely, we observe an improvement in scores on the K562 dataset because now the GRCh38 K562 Hi-C dataset has a sequencing depth more similar to the number of reads in the Hg19 Hi-C contact map (1.9 billion vs. 2.2 billion), and this similarity of feature density in both contact maps manifests as improvement in scores. There are distributional differences in both Hg19 assembled Hi-C contact maps, and GRCh38 assembled contact maps that we plan to investigate in more detail as part of our future work. We have also released the GrapHiC weights trained for GRCh38 model available.

	MSE	SSIM	PCC	HiCRep	GenomeDISCO	HiCSpector	QuASAR-Rep	TAD Boundaries	Chromatin Loops	DNA Hairpins
**graphic-basic**	0.00181278963	0.838731225	0.9556718263	0.3561238127	0.6889723173	0.2561238791	0.6187238192	0.456128371	0.1251243891	0.101512812
**graphic-pos**	0.001040138886	0.9118280711	0.9744101287	0.8011612903	0.8011612903	0.4349677419	0.8416831613	0.599158595	0.4393064431	0.4483152259
**graphic-ctcf**	0.000885409594	0.923217525	0.9793579727	0.831483871	0.8572446452	**0.4888709677**	0.8572446452	0.6730900735	0.5416144993	0.5285637621
**graphic**	**0.0008290408296**	**0.9239148305**	**0.9805694712**	0.8364516129	**0.9089642857**	0.4707096774	0.8614972581	0.6757557069	**0.5484932834**	**0.5475020008**
**graphic-large**	0.0008437751676	0.9226424879	0.9802531959	**0.8576774194**	0.8943636364	0.4867419355	**0.8756502903**	**0.7003308268**	0.5374315339	0.5385440433

TABLE S4
This table shows detailed ablations results on the GM12878-LRC-1 Hi-C dataset.

	MSE	SSIM	PCC	HiCRep	GenomeDISCO	HiCSpector	QuASAR-Rep	TAD Boundaries	Chromatin Loops	DNA Hairpins
**graphic-basic**	0.00171278963	0.8461231251	0.955657121	0.3912381273	0.7081230912	0.263891273	0.6571283012	0.4981237819	0.2025871212	0.179289931
**graphic-pos**	0.0009828922339	0.9128129021	0.9760758355	0.783516129	0.8600357143	0.4607419355	0.8362809677	0.6173654048	0.4367204234	0.4443665519
**graphic-ctcf**	0.0008680545725	0.9223760309	0.9798637377	0.7907096774	0.8942413793	**0.4751612903**	0.8521077742	0.6845039088	0.5351110329	0.5324476257
**graphic**	**0.0008289036923**	**0.9228198434**	**0.9805673792**	0.8179032258	**0.9030384615**	0.457	0.8575350323	0.6821782829	**0.5365461676**	**0.5487044283**
**graphic-large**	0.000847046962	0.922221218	0.9803199076	**0.8322580645**	0.8888636364	0.4499032258	**0.8671765161**	**0.6968594479**	0.5194946749	0.5372154113

TABLE S5
This table shows detailed ablations results on the GM12878-LRC-2 Hi-C dataset.

	MSE	SSIM	PCC	HiCRep	GenomeDISCO	HiCSpector	QuASAR-Rep	TAD Boundaries	Chromatin Loops	DNA Hairpins
**graphic-basic**	0.0017591283	0.8451236123	0.9563819028	0.4015812312	0.7123019823	0.268317541	0.6667238192	0.5123871251	0.2312873196	0.191512812
**graphic-pos**	0.0009522660403	0.9186408093	0.9774438178	0.8046774194	0.8921034483	0.4806129032	0.8345150968	0.5999143664	0.4356771671	0.39638861
**graphic-ctcf**	0.0009	0.9222859577	0.9793420255	0.8116451613	0.9049285714	**0.4852903226**	0.8512654516	0.6683	0.5164511455	0.5167336452
**graphic**	0.0008672421682	0.9224354332	0.9797065909	0.8007741935	**0.91**	0.4613548387	0.8608621613	0.6647960639	**0.5216039742**	**0.52716**
**graphic-large**	**0.0008528126054**	**0.9224282913**	**0.9801922214**	**0.8458709677**	0.8855833333	0.4707741935	**0.8617122903**	**0.6885320461**	0.5096508442	0.525595418

TABLE S6
This table shows detailed ablations results on the GM12878-LRC-3 Hi-C dataset.

	MSE	SSIM	PCC	HiCRep	GenomeDISCO	HiCSpector	QuASAR-Rep	TAD Boundaries	Chromatin Loops	DNA Hairpins
**graphic-basic**	0.00171278591	0.8356182415	0.9551293856	0.3981293789	0.7032179831	0.2695812031	0.6538921731	0.5021987451	0.2212873126	0.171512812
**graphic-pos**	0.0009809146868	0.9193714093	0.9764888496	0.7997096774	0.8847586207	0.4398064516	0.838564129	0.5726642243	0.3868603552	0.4073110319
**graphic-ctcf**	0.0009762486443	0.9200183289	0.9776983314	0.8104193548	0.8972068966	**0.4779354839**	0.8548853548	0.6748089642	0.5089955344	0.5018595278
**graphic**	0.0009478544234	**0.920667914**	0.9779336097	0.8148064516	**0.9058461538**	0.4660322581	0.8636027333	0.6639213627	**0.5150424406**	**0.5337003302**
**graphic-large**	**0.0008896560175**	0.9204220266	**0.9792669186**	**0.8453225806**	0.88308	0.4606451613	**0.8677272903**	**0.6812338361**	0.4997126192	0.5250535956

TABLE S7
This table shows detailed ablations results on the GM12878-LRC-4 Hi-C dataset.

	MSE	SSIM	PCC	HiCRep	GenomeDISCO	HiCSpector	QuASAR-Rep	TAD Boundaries	Chromatin Loops	DNA Hairpins
**graphic-basic**	0.00201278963	0.828128741	0.9467128312	0.3017892319	0.625198231	0.2561238791	0.5517238112	0.430897451	0.1181023712	0.101512812
**graphic-pos**	0.001829135232	0.8873864556	0.9519068669	0.4235806452	0.7322068966	0.276516129	0.7074913548	0.3248079352	0.155861061	0.1206382959
**graphic-ctcf**	0.001466627116	0.8959276496	0.9661399373	0.5114516129	0.75724	0.2982258065	0.7384491667	0.4848169158	0.3012013986	0.3201851551
**graphic**	0.001204534899	0.9005296682	0.9698582723	0.5322903226	0.8051363636	0.3179677419	0.7461324138	0.5386603403	0.3410283165	0.3874506211
**graphic-large**	**0.001106260577**	**0.902333818**	**0.9728707875**	**0.5829677419**	**0.8374375**	**0.3242903226**	**0.7815497333**	**0.5535101466**	**0.3868443661**	**0.4281288537**

TABLE S8
This table shows detailed ablations results on the GM12878-LRC-5 Hi-C dataset.

		MSE	SSIM	PCC	HiCRep	GenomeDISCO	HiCSpector	QuASAR-Rep	TAD Boundaries	Chromatin Loops	DNA Hairpins
**GM12878-LRC-1**	**HiCReg**	0.00525	0.73768	0.86519	0.37990	0.63565	0.24605	0.68039	0.41440	0.18041	0.22014
	**HiCNN**	0.00150	0.90890	0.97770	**0.90182**	0.80220	0.46291	**0.87124**	**0.71041**	0.53060	0.52280
	**GrapHiC**	**0.00083**	**0.92391**	**0.98057**	0.83645	**0.90896**	**0.47071**	0.86150	0.67576	**0.54849**	**0.54750**
**GM12878-LRC-2**	**HiCReg**	0.00525	0.73768	0.86519	0.37990	0.63565	0.24605	0.68039	0.41440	0.18041	0.22014
	**HiCNN**	0.00135	0.92020	0.97455	0.80125	**0.91780**	0.45215	0.00000	**0.71103**	0.51860	0.54050
	**GrapHiC**	**0.00083**	**0.92282**	**0.98057**	**0.81790**	0.90304	**0.45700**	**0.85754**	0.68218	**0.53655**	**0.54870**
**GM12878-LRC-3**	**HiCReg**	0.00525	0.73768	0.86519	0.37990	0.63565	0.24605	0.68039	0.41440	0.18041	0.22014
	**HiCNN**	0.00414	0.83110	0.92730	0.79512	0.82610	0.45613	0.00000	**0.72110**	0.52150	0.51940
	**GrapHiC**	**0.00087**	**0.92244**	**0.97971**	**0.80077**	**0.91000**	**0.46135**	**0.86086**	0.66480	**0.52160**	**0.52716**
**GM12878-LRC-4**	**HiCReg**	0.00525	0.73768	0.86519	0.37990	0.63565	0.24605	0.68039	0.41440	0.18041	0.22014
	**HiCNN**	0.00456	0.81420	0.91770	0.79124	0.79340	0.46215	0.00000	**0.70990**	0.50560	0.45070
	**GrapHiC**	**0.00095**	**0.92067**	**0.97793**	**0.81481**	**0.90585**	**0.46603**	**0.86360**	0.66392	**0.51504**	**0.53370**
**GM12878-LRC-5**	**HiCReg**	0.00525	0.73768	0.86519	0.37990	0.63565	0.24605	0.68039	0.41440	0.18041	0.22014
	**HiCNN**	0.00809	0.68010	0.83977	0.47193	0.47380	0.28129	0.00000	0.43825	0.22160	0.15606
	**GrapHiC**	**0.00120**	**0.90053**	**0.96986**	**0.53229**	**0.80514**	**0.31797**	**0.74613**	**0.53866**	**0.34103**	**0.38745**

TABLE S9
We show the performance of GrapHiC when provided with five different sparse GM12878 datasets. We compare the performance of GrapHiC against HiCReg, HiCNN and bold score of the best performing method.

		MSE	SSIM	PCC	HiCRep	GenomeDISCO	HiCSpector	QuASAR-Rep	TAD Boundaries	Chromatin Loops	DNA Hairpins
IMR90	**HiCReg**	0.05150	0.51890	0.26980	0.13090	0.18240	0.10670	0.17740	0.35420	0.15290	0.08620
	**HiCNN**	**0.00510**	0.65370	0.56760	**0.80790**	0.51510	0.24840	0.00000	**0.56030**	0.29290	0.26730
	**GrapHiC**	0.00750	**0.78031**	**0.80270**	0.69055	**0.61493**	**0.28152**	**0.77327**	0.55940	**0.47150**	**0.47480**
K562	**HiCReg**	0.05656	0.54135	0.48524	0.27575	0.36215	0.15900	0.51221	0.42812	0.19952	0.27708
	**HiCNN**	0.01100	0.64760	0.61870	**0.87120**	0.77460	0.29510	0.00000	**0.58190**	0.35720	0.26500
	**GrapHiC**	**0.00470**	**0.82780**	**0.87310**	0.70884	**0.84704**	**0.32516**	**0.73423**	0.53880	**0.45220**	**0.37030**

TABLE S10
We show the performance of GrapHiC when provided with two different cell line datasets. We compare the performance of GrapHiC against HiCReg, HiCNN and bold score of the best performing method.

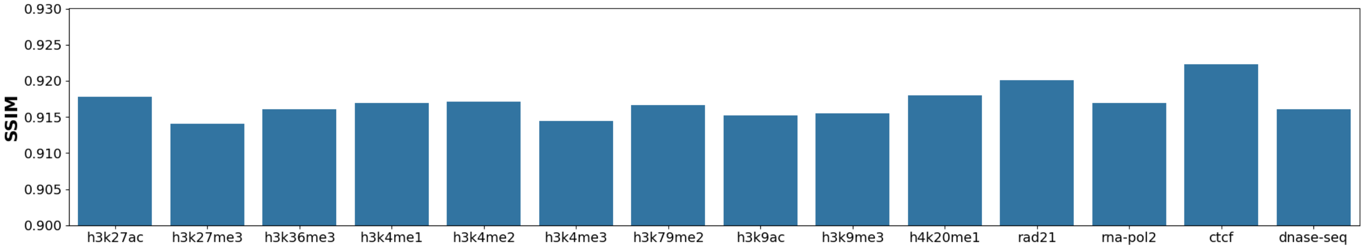

Fig. S1. We design an experiment to find which ChIP-seq by itself provides achieves the best performance in predicting Hi-C contact maps. We compare the performance on all fourteen ChIP-seq signals used by HiCReg and we find that CTCF ChIP-seq provides best predictive performance.

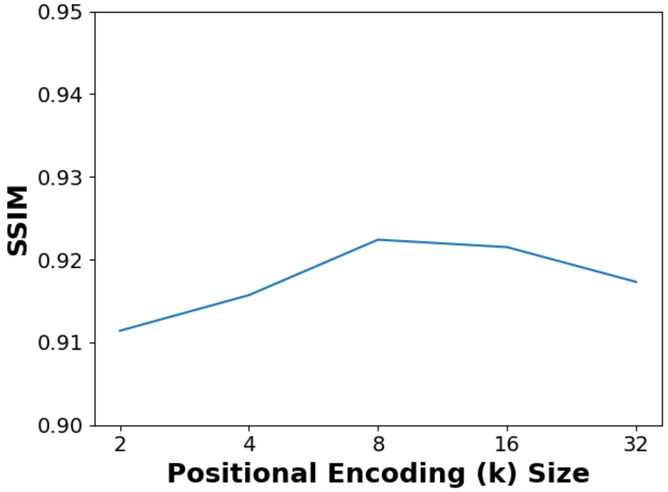

Fig. S2. We design an experiment to test how the size of positional encoding "k" affect the performance of GrapHiC. We test positional encodings in the range of two to thirty-two. We through our evaluations find that GrapHiC acheives best score when $k = 8$.

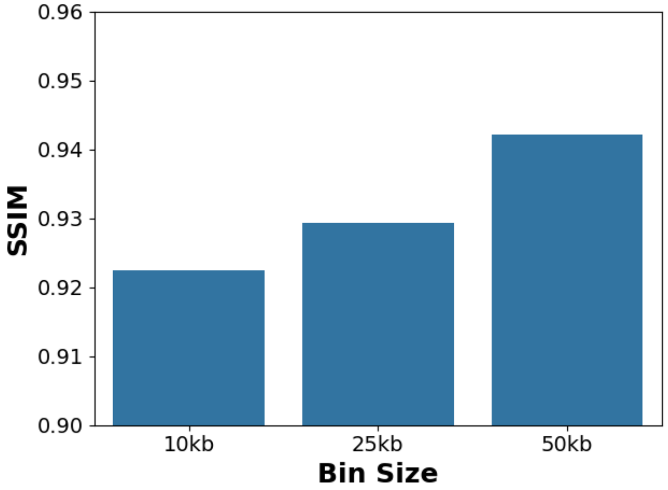

Fig. S3. We design an experiment to test whether GrapHiC can generalize to other bin sizes. We retrain and test the performance of GrapHiC with 25Kbp and 50Kbp bin size and compare their scores against 10Kbp. We find that GrapHiC scores (on SSIM metric) improve as we increse the bin size suggesting that GrapHiC can generalize to larger bin sizes as well.

	MSE	SSIM	PCC	HiCRep	GenomeDISCO	HiCSpector	QuASAR-Rep	TAD Boundaries	Chromatin Loops	DNA Hairpins
**GM12878**	0.00110	0.90830	0.97220	0.59400	0.84428	0.33185	0.77946	0.61640	0.36520	0.44350
**IMR90**	0.00490	0.77460	0.85050	0.35865	0.57363	0.24410	0.63696	0.54500	0.37280	0.40060
**K562**	0.00440	0.82800	0.87410	0.47852	0.80279	0.28448	0.63149	0.51250	0.33050	0.31200

TABLE S11
We show the performance of GrapHiC when provided with an expected Hi-C contact map across three different cell lines.

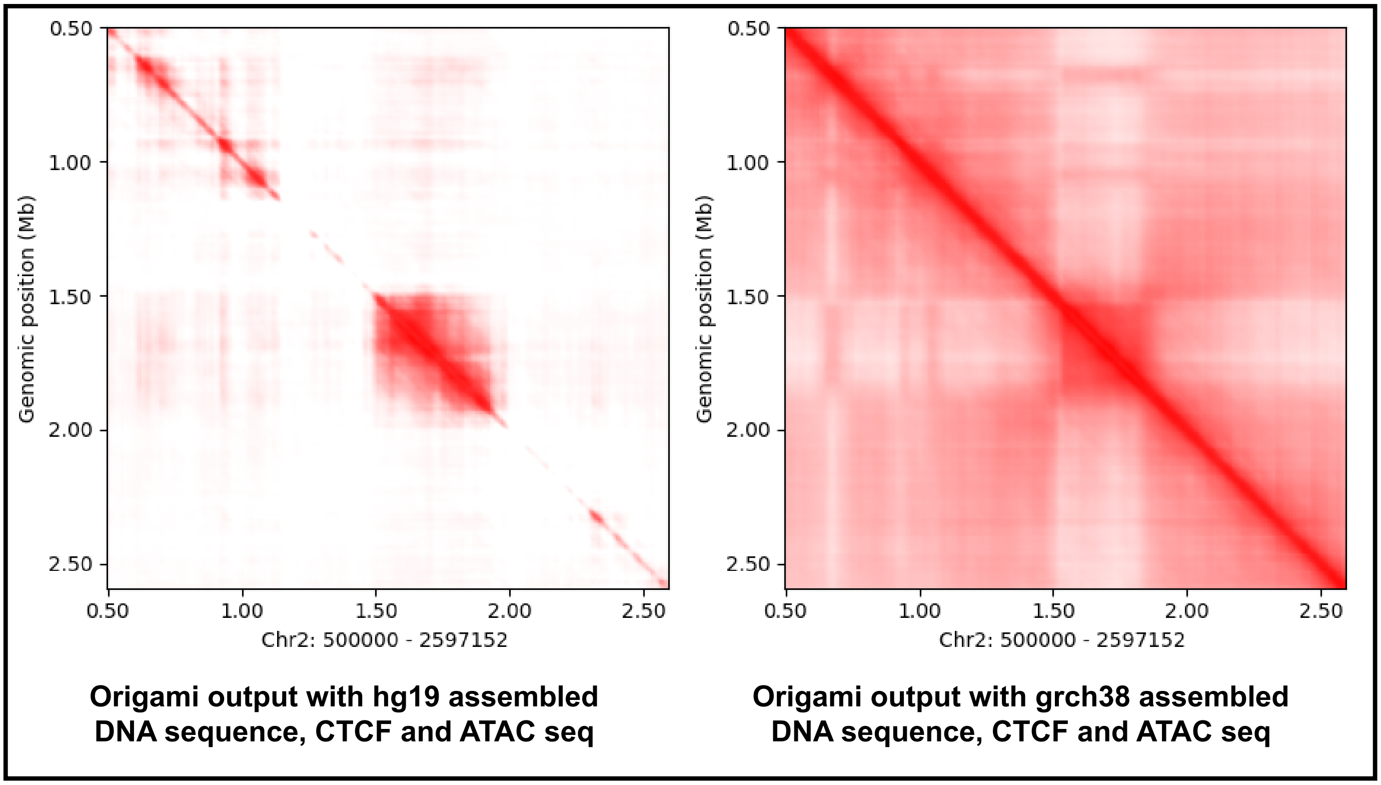

Fig. S4. We compare the output of Origami, with inputs aligned on hg19 genome assembly against the grch38 aligned assembly. Origami does not generalize to hg19 assembled inputs and struggles to produce meaningful contact maps.

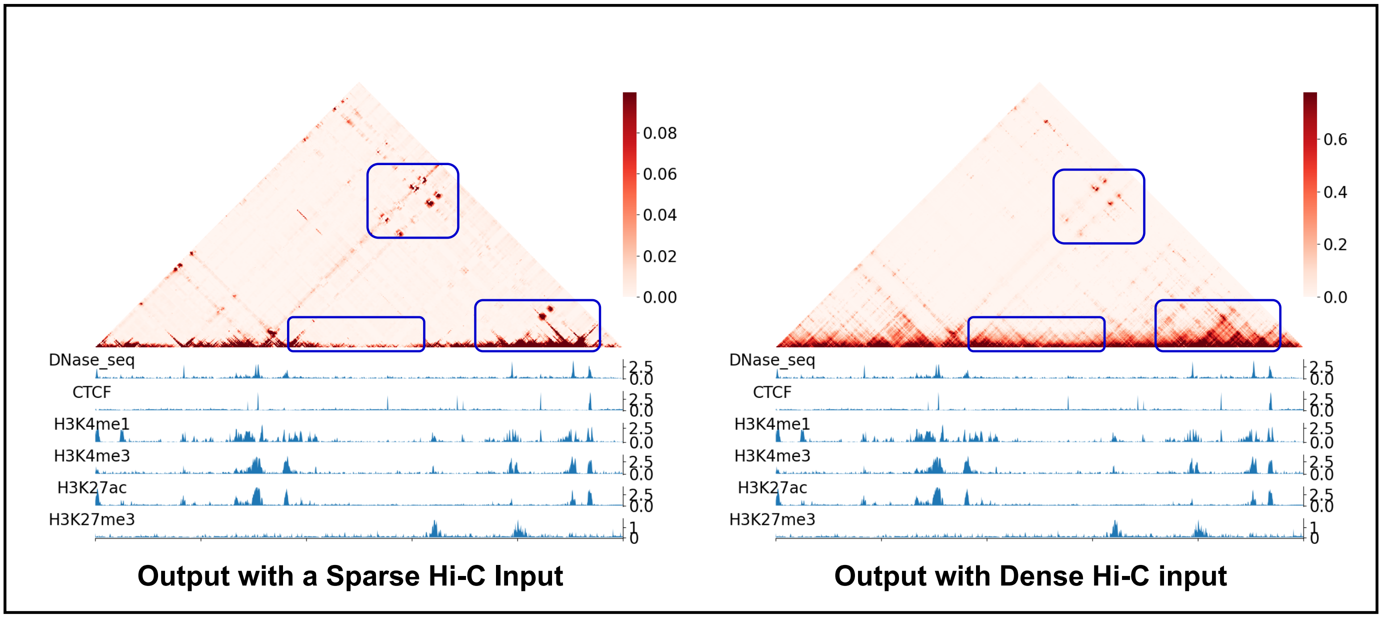

Fig. S5. We compare the output of Caesar when provided with a sparse real-world H1 cell line Hi-C contact map as input. Caesar struggles to recover distal and nearby features to the diagonal. We provided the same ChIP-seq inputs in both cases. Moreover, Caesar produces MicroC contact maps showing substantially different read contact distributions compared to Hi-C, so we exclude Caesar from our baselines.

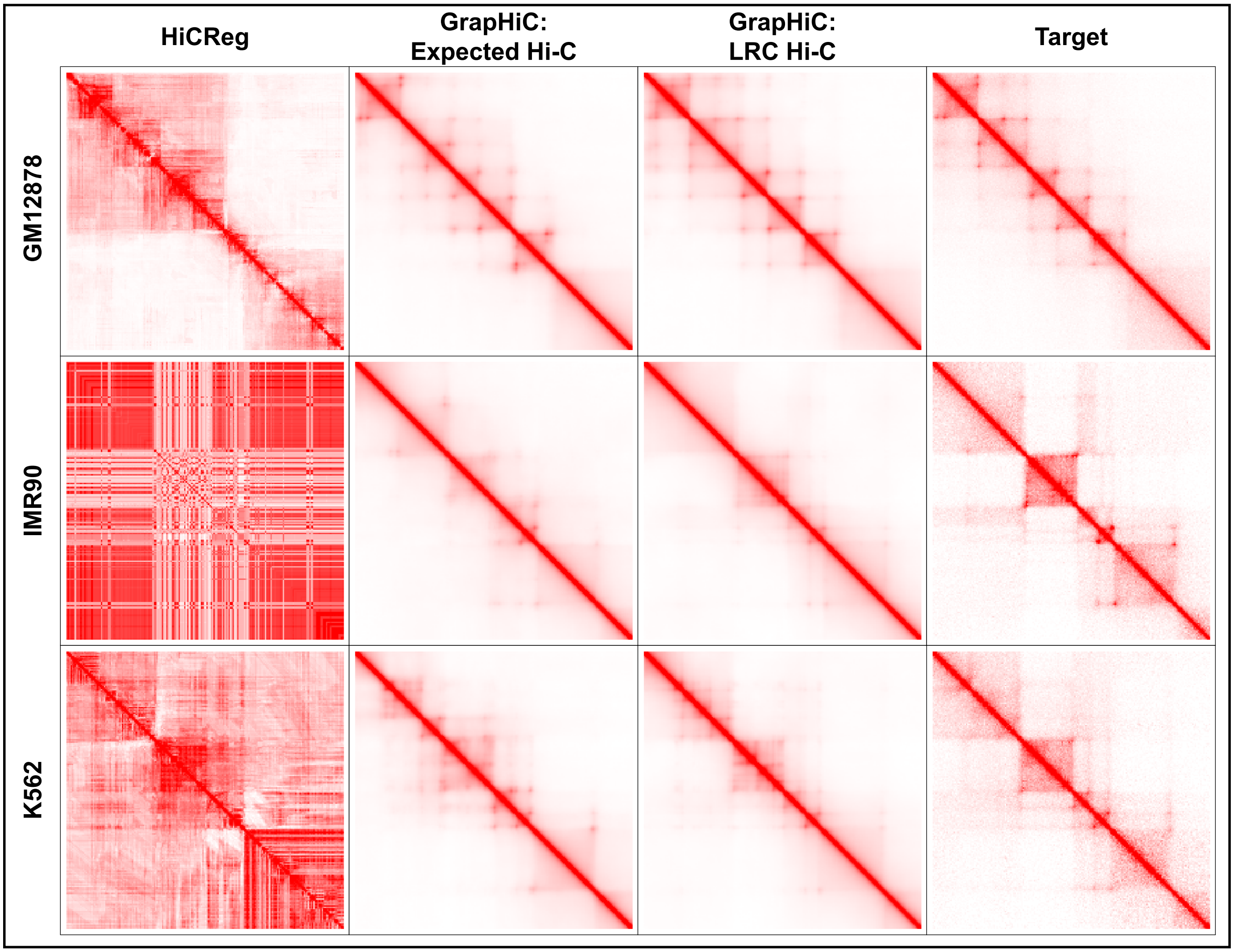

Fig. S6. We qualitatively compare the output of GraphiC when provided with a expected Hi-C contact map, a low-read-count (LRC) contact map against the target and HiCReg. We show that GraphHiC is able to impute high-fidelity Hi-C contact maps in both cases that are more simlar to the target in comparison to HiCReg.
